# The sharp edge of immunosuppressive treatments: infections

**DOI:** 10.55730/1300-0144.5845

**Published:** 2024-05-07

**Authors:** Aybegüm ÖZŞAHİN, Tuba İLGAR, Sudem MAHMUTOĞLU ÇOLAK, Kübra AKYÜZ, Melih Gaffar GÖZÜKARA, Uğur KOSTAKOĞLU, İlknur Esen YILDIZ, Ayşe ERTÜRK

**Affiliations:** 1 Department of Infectious Diseases and Clinical Microbiology, Recep Tayyip Erdoğan University Training and Research Hospital, Rize, Turkiye; 2 Department of Infectious Diseases and Clinical Microbiology, Faculty of Medicine, Recep Tayyip Erdoğan University, Rize, Turkiye; 3 Department of Pulmonary Diseases, Üsküdar State Hospital, İstanbul, Turkiye; 4 Department of Public Health, Faculty of Medicine, Ankara Yıldırım Beyazıt University, Ankara, Turkiye

**Keywords:** Anticytokine, anakinra, tocilizumab, infection, COVID-19

## Abstract

**Background and aim:**

Different side effects, including infections, are encountered in patients receiving anticytokines used for the treatment of severe coronavirus disease 2019 (COVID-19). The aim of this study was to evaluate the infections and the effects of these infections that develop in this patient group.

**Materials and Methods:**

This study included 208 patients who were followed-up with the diagnosis of severe COVID-19 in two different hospitals. Patient data were obtained retrospectively from the hospital information system.

**Results:**

Of the 208 patients included, 54 were in the anakinra group, and 154 were in the tocilizumab group. Of these patients, 73 (35.1%) developed infection, 160 (76.9%) were admitted to the intensive care unit (ICU), and the 30-day mortality rate was 46.6%. The ICU admission, 30-day mortality, and infection rates were higher in the anakinra group, but it was not statistically significant (p = 0.137, p = 0.127, and p = 0.132, respectively), while pneumonia and bloodstream infection (BSI) rates were higher (p = 0.043 and p = 0.010 respectively). The 30-day mortality rate was significantly higher in patients who developed infection, especially in the tocilizumab group (p < 0.001 and p = 0.001). The independent risk factors affecting the development of infection were evaluated via regression analysis, in which it was found that age, sex, and the type of immunosuppressive treatment had no significant effect, while ICU admission increased the risk of infection by 32.8 times (95% CI: 4.4–245.8) and each day of hospitalization slightly increased the risk of infection by 1.06 times (95% CI: 1.03–1.09).

**Conclusion:**

Infection rates were higher in the anakinra group, especially the pneumonia and BSI rates were higher than in the tocilizumab group. The 30-day mortality rates were higher in patients who had an infection, especially in the tocilizumab group. This is one of the rare studies that evaluated infections developing in patients treated with anakinra and tocilizumab together.

## 1. Introduction

Immunosuppressive therapies have long been used to treat hyperinflammation caused by autoimmune diseases or infections. The oldest known immunosuppressive drugs are corticosteroids, and new treatments, which are more effective and have fewer side effects, are beginning to be used every day. These molecules, which can be beneficial if used appropriately, can also have severe side effects, including death. This has been encountered quite frequently with the immunosuppressive drugs used in the treatment of coronavirus disease 2019 (COVID-19), which has affected the world for the past 4 years.

Severe acute respiratory syndrome coronavirus 2 (SARS-CoV-2), which is the causative agent of COVID-19, enters cells by attaching to the ACE-2 receptor within the cells, initiating the replication cycle, while also triggering the innate and adaptive immune responses, causing a cytokine storm and uncontrolled hyperinflammation. Various cytokines, chemokines, and immune cells are activated during this cytokine storm, particularly macrophages. Studies have shown that the most important factor causing serious disease is the uncontrolled and excessive immune response of the host [1,2]. As a result, pneumonia, organ damage, acute respiratory distress syndrome (ARDS), and in some cases, mortality develop.

Macrophage activation syndrome (MAS) is a condition caused by a cytokine storm and characterized by hyperferritinemia and coagulopathy. It is thought that COVID-19-related immune exhaustion or defective antiviral response cause this syndrome. For this reason, studies have been conducted to use antiinterleukin 1 (IL-1) and IL-6 agents used in the treatment of MAS for the treatment of severe COVID-19 cases [3]. It has been shown that immunosuppressive and anticytokine treatments, at the right time, in the right doses, and correctly selected, increase survival in the treatment of hyperinflammatory response [4–6]. Hence, the COVID-19 treatment guidelines suggest administering corticosteroids to hospitalized, hypoxic patients and considering immunomodulatory therapies like anakinra (Kineret) or tocilizumab (Actemra) for individuals who do not show reduced oxygen requirements or systemic inflammatory response improvement following steroid treatment [6–10]. It is known that these drugs, which are used to reduce morbidity and mortality, may cause side effects such as application-related local reactions, secondary infections, hypertension, disorders in liver function tests, gastrointestinal bleeding, pulmonary embolism, and even intestinal perforation [6,11–13].

The current research sought to investigate the occurrence rate of infections, the pathogens responsible for these infections, and the impact this has on mortality among patients being treated with anakinra or tocilizumab. These medications were commonly utilized during the pandemic and are anticipated to remain key in managing hyperinflammation following autoimmune diseases or infections.

## 2. Materials and methods

Patients over the age of 18, who were followed-up between March 1st, 2020, and December 31st, 2021, with a diagnosis of COVID-19 at the 2nd level state hospital and 3rd level university hospital in our city, who received anakinra or tocilizumab with the diagnosis of MAS due to COVID-19, were included in the study. Patients were examined in 2 groups as the anakinra or tocilizumab groups. Patient data such as age, sex, immunosuppressive treatment received, intensive care unit (ICU) follow-up, presence of 30-day mortality, length of hospital stay, infections that developed after receiving immunosuppressive treatment, the causative agents of these infections, and the effect of infection development on mortality were retrospectively scanned from the hospital data system. Only the infections in which the agent could be isolated in blood, sputum, tracheal aspirate, and urine sample cultures were included. Bloodstream infections (BSIs) secondary to another infection focus, sequential culture positivities, asymptomatic candiduria, and culture results evaluated in favor of colonization were excluded.

### 2.1. Treatment administration and selection

All hypoxic patients who were hospitalized with the diagnosis of COVID-19 were started on standard dose or high-dose corticosteroid treatments. Patients who failed to improve with first-line treatment were evaluated for MAS and the need for anticytokine therapy.

MAS was diagnosed according to the COVID-19 treatment guide of the Ministry of Health of the Republic of Türkiye. Patients with resistant fever, ongoing elevation of C-reactive protein, ferritin level above normal or continued to increase, elevated D-dimer levels, lymphopenia, neutrophilia, thrombocytopenia, and deterioration in liver function tests were evaluated for MAS [14]. Anticytokine therapy was started in patients whose procalcitonin levels were negative and who had no evidence of secondary infection according to clinical evaluation. Tocilizumab was administered as an intravenous infusion at a dose of 8 mg/kg in two consecutive doses, while anakinra was started at a dose of 2–10 mg/kg (subcutaneous) and discontinued by reducing the dose according to the patients’ condition. The choice of tocilizumab or anakinra was made based on the physician’s preference and the availability of the drugs.

### 2.2. Ethics committee approval

Approval was obtained from the ethics committee of Recep Tayyip Erdoğan University, where the preresearch study was carried out, with decision number 2023/54, dated 23/2/23.

### 2.3. Statistical analysis

The statistical analysis was performed with IBM SPSS Statistics for Windows 24.0 (IBM Corp., Armonk, NY, USA). The distribution of the data was checked using visual (histogram) and Kolmogorov–Smirnov tests. The age and length of hospital stay variables were distributed nonparametrically. In the presentation of the data, numbers (n), percent (%), and the median with minimum–maximum values were used. Pearson’s chi-squared test and Fisher’s Exact test were used in the statistical analysis of the categorical data. The Mann–Whitney U test was used for the parametric comparison of the numerical data of the 2 groups. Binomial regression was used to determine the factors that affected infection diagnosis. Statistical significance was accepted as p < 0.05 at a 95% confidence interval (CI).

## 3. Results

**Figure 1:**
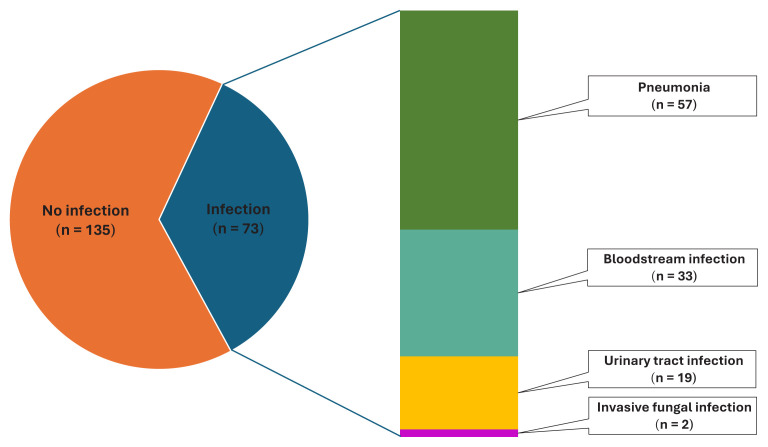
Infections developing in patients receiving immunosuppressive therapy.

**Figure 2:**
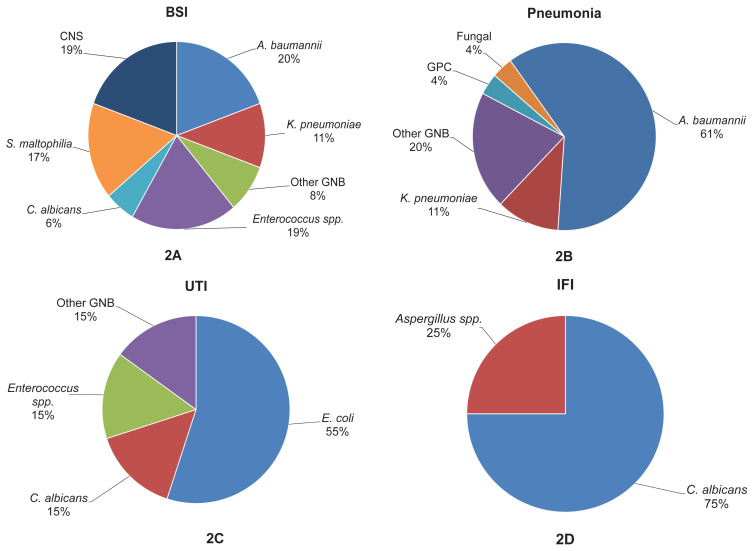
Causative agent distribution of BSI (**2A**), pneumonia (**2B**), UTI (**2C**), and IFI (**2D**). **2A** BSI: Bloodstream infection, CNS: coagulase negative staphylococci, other GNB: *E. coli, B. cepacian*. **2B** Fungal infections: *C. albicans* and *Aspergillus* spp.; GPC: *Enterococcus* spp., *S. pneumoniae*, and other GNB: *E. coli*, *P. aeruginosa*, *K. pneumoniae*, *S. maltophilia*, *Enterobacter* spp., and *B. cepacia*. **2C** UTI: Urinary tract infection and other GNB: *K. pneumoniae* and *P. aeruginosa*. **2D** IFI: Invasive fungal infection.

A total of 208 patients were included in the study in 2 groups, with 54 patients in the anakinra group, and 154 patients in the tocilizumab group. All the patients receiving anakinra or tocilizumab had received standard-dose or pulse-dose steroids simultaneously or before it. Of the patients, 79 (38%) were female, with a median age of 63.5 (range: 24–94) years, and a length of hospital stay of 18 (range: 6–75) days. There were 160 (76.9%) patients admitted to the ICU, and the 30-day mortality was 46.6%. Secondary infection developed during hospitalization in 73 patients (35.1%) (Figure 1). BSIs developed in 33 (15.9%) patients, and three patients had more than one BSI attack. Pneumonia developed in 57 (27.4%) patients, seven of whom had more than one pneumonia attack. Urinary tract infections developed in 19 (9.1%) patients and two (1%) developed invasive fungal infections (IFIs). The most frequently identified microorganisms in the BSIs were gram-positive cocci, (*Enterococcus* spp. and coagulase negative streptococci), followed by *Acinetobacter A. baumannii* and *Stenotrophomonas maltophilia*. *A. baumannii* was also the leading causative agent in pneumonia. In the urinary tract infections, *Escherichia coli* was the most frequently isolated microorganism. *Candida albicans* was the most common agent identified in the IFIs. The distribution of causative agents is shown in Figure 2.

Since admission to ICU was thought to be a risk factor for the development of infection, this patient group was evaluated separately. Of these160 patients, 65 (40.6%) were female, and the median age was 64 (range: 24–88) years. In regard to treatment, 46 (28.7%) received anakinra, and 114 (71.3%) received tocilizumab. Moreover, 72 (45%) developed an infection during hospitalization, and the 30-day mortality rate was 58.1%.

When all patients in the two groups were evaluated, there was no difference in the mean ages and rates of ICU admission, 30-day mortality, secondary infection, UTI, and IFI values. The ICU admission, mortality, and infection development rates were higher in the anakinra group than in the tocilizumab group, but this was not statistically significant. Pneumonia and BSI rates in the anakinra group were higher than in the tocilizumab group (p = 0.043 and p = 0.010, respectively). In the anakinra group, the average length of hospital stay was lower (p = 0.046). When the patients followed-up in the ICU were evaluated separately, no statistically significant relationship was found between the groups in terms of the 30-day mortality and infection development rates (p = 0.533 and p = 0.326, respectively). The BSI rate was significantly higher in the anakinra group than in the tocilizumab group (p = 0.021) (Table 1).

Factors affecting the development of infection were evaluated, and no significant difference was found in terms of sex or median age (p = 0.496 and p = 0.715, respectively). The rate of infection in patients admitted to the ICU was significantly higher (p < 0.001). Among all the patients and patients admitted to the ICU, the median length of hospital stay in patients who developed infection was longer than in those who did not (p < 0.001 and p = 0.009, respectively) (Table 2).

The 30-day mortality rate in patients who developed infection was significantly higher than in those who did not (p < 0.001). When the treatment groups were evaluated separately in terms of the 30-day mortality rate, there was no significant relationship between the anakinra group (p = 0.141) and in the tocilizumab group. The rate was significantly higher in patients who developed infection than in those who did not (p = 0.001) (Table 3).

Independent risk factors that were thought to affect the development of infection in patients diagnosed with COVID-19 and receiving immunosuppressive treatment were evaluated by regression analysis. The odds ratio (OR) of infection among all the patients increased by 1.048 times (95% CI: 1.015–1.082) for each day of hospitalization, and by 32.819 times (95% CI: 4.382–245.821) for ICU admission. For patients admitted to the ICU, each day’s increase in the length of stay increased the probability of infection by 1.048 times (95% CI: 1.015–1.082) (Table 4). The immunosuppressive treatments received by the patients in both groups did not increase the risk of infection.

## 4. Discussion

Unlike previous reviews that analyzed similar topics, this article is one of the rare studies in which infections developed in patients who received anakinra and tocilizumab were evaluated together. In the anakinra group, the ICU admission, 30-day mortality, and infection rates were lower but not statistically significant, whereas the pneumonia and BSI development rates were significantly higher than in the tocilizumab group (p = 0.137, p = 0.127, p = 0.132, p = 0.043, and p = 0.01, respectively). Development of an infection increased the 30-day mortality rate in all the patients, especially in the tocilizumab group (p = 0.001 and p = 0.001 respectively). ICU admission and length of stay were independent risk factors for the development of infection.

Suppressing hyperinflammation is one of the building blocks of COVID-19 treatment. Studies have been conducted on the effectiveness and side effects of anticytokine treatments such as anakinra, tocilizumab, sarilumab, and canakinumab [7,9,10,15,16].

Anakinra, an IL-1 receptor antagonist, has been used to treat hyperinflammation and MAS caused by COVID-19 [17]. In a systematic review evaluating serious infections in patients using biological agents, the rate of severe infections was 5.1%, and pneumonia (23.8%) was the most common infection in the anakinra group [12]. In another study comparing anakinra and standard treatment in COVID-19 patients, 26.8% of the patients in the anakinra group had infections (8% BSIs, 3.6% pneumonia) and 3.6% of these patients died. There was no statistically significant difference between the groups [10]. In the present study 44.4% of the patients in the anakinra group developed an infection, of whom 38.9% had pneumonia and 27.8% had BSIs. The higher infection rates in the current study group may have been due to the high rate of ICU admission and mortality, which may show that the study group had more severe conditions. In addition, the fact that all the patients received steroids simultaneously may have caused their immune systems to be more suppressed. The reason for the higher pneumonia rates was that the patients likely already had damaged lung tissues due to COVID-19 pneumonia.

Tocilizumab is a competitive inhibitor of the receptor for IL-6, a cytokine with pro and antiinflammatory effects [18,19]. In the randomized, controlled, REMAP-CAP and RECOVERY studies, which evaluated the effectiveness of IL-6 receptor antagonists in the treatment of COVID-19, the secondary infection rates ranged from 0.07% to 0.3%. Moreover, the secondary infection rates ranged from 14.2% to 40.4% in different cohorts [20,21]. Of the patients included in the present study who received tocilizumab, 74% were admitted to the ICU, the mortality rate was 56.5%, and 31.8% developed an infection. The infection rates were higher than in randomized controlled trials, but similar to those of other cohorts. The reason for this high rate may be the very high ICU admission rates and the patients’ prior use of corticosteroids.

Looking at studies that have compared side effects of immunosuppressive treatments, in a review including 3073 patients receiving anticytokine therapy and 6572 patients as the control group that evaluated the effectiveness of these treatments and secondary infections in COVID-19 patients, anticytokine therapy did not increase the infection rate. The infection rate was higher in the anakinra group (OR: 1.44, 95% CI: 0.47–4.43, p = 0.520) compared to the tocilizumab group (OR: 1.12,95% CI: 0.87–1.43, p = 0.376), but it was not statistically significant [22]. In another study including 235 patients that compared anakinra and tocilizumab, secondary infection rates were similar between the groups (6.3% vs. 9.2%, p = 0.44). Moreover, the 28-day mortality rates and ICU admission rates were similar (p = 1 and p = 0.30, respectively) [23].

In the current study, secondary infections caused an increase in the 30-day mortality rates, especially in the tocilizumab group. In a study evaluating factors affecting mortality in COVID-19, secondary bacterial coinfections in patients receiving tocilizumab were associated with mortality (p = 0.002) [24]; however, there are also studies that have found the opposite [25].

The limitations of this study were that it was retrospective, the initial clinical conditions of the patients were not known, and there was no control group. The strength of the study was that it is one of the rare studies in which both treatment groups were examined together, the developing infections and infectious agents were analyzed in detail, and the effect of infection development on mortality was examined. Randomized controlled studies on this subject are needed to obtain clearer data.

## 5. Conclusion

Although the pandemic is over and the number and severity of cases have decreased, the anticytokines used in the treatment of COVID-19 will continue to be used in the treatment of hyperinflammatory syndrome and MAS that develop due to infections or rheumatological diseases. It should be kept in mind that infections may develop as a side effect of anakinra and tocilizumab, and especially anakinra has a higher risk in this regard. Treatment selection and patient follow-up should be shaped accordingly. Prospective, randomized studies are needed to further elucidate this issue.

## Figures and Tables

**Table 1:** Distribution of patients according to the treatments they received, sex, need for intensive care, and infection rates.

			Anakinra	Tocilizumab	p-value
All patients (n = 208)	Sex (n/%*)				
		Female	29 (53.7)	50 (32.5)	**0.006** #
		Male	25 (46.3)	104 (67.5)	
	Age (years)**		66 (27–94)	63 (24–88)	0.650¥
	Length of hospital stay (days)**		17 (6–61)	21 (7–75)	**0.046** ¥
	ICU (n/%*)				
		No	8 (14.8)	40 (26.0)	0.137#
		Yes	46 (85.2)	114 (74.0)	
	30-day mortality (n/%*)				
		No	24 (44.4)	87 (56.5)	0.127#
		Yes	30 (55.6)	67 (43.5)	
	Infection (n/%*)				
		No	30 (55.6)	105 (68.2)	0.132#
		Yes	24 (44.4)	49 (31.8)	
	Pneumonia (n/%*)				
		No	33 (61.1)	118 (76.6)	**0.043** #
		Yes	21 (38.9)	36 (23.4)	
	BSI (n/%*)				
		No	39 (72.2)	136 (88.3)	**0.010** #
		Yes	15 (27.8)	18 (11.7)	
	UTI (n/%*)				
		No	47 (87.0)	142 (92.2)	0.277#
		Yes	7 (13.0)	12 (7.8)	
	IFI (n/%*)				
		No	54 (100)	152 (98.7)	1.000
		Yes	0 (0.0)	2 (1.3)	
Patients followed-up in the ICU (n = 160)	30-day mortality (n/%*)				
		No	17 (37.0)	50 (43.9)	0.533#
		Yes	29 (63.0)	64 (56.1)	
	Infection (n/%*)				
		No	22 (47.8)	66 (57.9)	0.326#
		Yes	24 (52.2)	48 (42.1)	
	Pneumonia (n/%*)				
		No	27 (58.7)	78 (68.4)	0.323#
		Yes	19 (41.3)	36 (31.6)	
	BSI (n/%*)				
		No	31 (67.4)	97 (85.1)	**0.021** #
		Yes	15 (32.6)	17 (14.9)	
	UTI (n/%*)				
		No	39 (84.8)	102 (89.5)	0.575#
		Yes	7 (15.2)	12 (10.5)	
	IFI (n/%*)				
		No	46 (100)	112 (98.2)	1.000
		Yes	0 (0.0)	2 (1.8)	

* Column percentage,

** Median (minimum–maximum),

# Pearson chi-squared test, and

¥ Mann–Whitney U test.

ICU: Intensive care unit, BSI: bloodstream infection, UTI: urinary tract infection, and IFI: invasive fungal infection.

**Table 2:** Factors affecting infection development.

			Infection		p-value
			No	Yes	
All patients (n = 208)	Sex (n/%*)				
		Female	49 (62.0)	30 (38.0)	0.496**
		Male	86 (66.7)	43 (33.3)	
	Age (years) (median [min – max])		64 (24 – 94)	63 (27 – 88)	0.715#
	Length of hospital stay (day) (median [min – max])		17 (6 – 54)	22 (9 – 75)	**<0.001** #
	ICU (n/%*)				
		No	47 (97.9)	1 (2.1)	**<0.001** **
		Yes	88 (55.0)	72 (45.0)	
Patients followed-up in the ICU (n = 160)	Sex (n/%*)				
		Female	35 (53.8)	30 (46.2)	0.135**
		Male	53 (55.8)	42 (44.2)	
	Age (year) (median [min – max])		65.5 (24 – 88)	63 (27 – 88)	0.483#
	Length of hospital stay (day) (median [min – max])		18 (6 – 54)	22 (9 – 75)	**0.009** #

* Percentage of rows,

** Pearson chi-squared test,

# Mann–Whitney U test.

**Table 3:** Effect of infection development on 30-day mortality.

		Infection (n/%*)		p-value
30-day mortality		No	Yes	
All patients (n = 363)				
	No	85 (63.0)	26 (35.6)	**<0.001** **
	Yes	50 (37.0)	47 (64.4)	
Anakinra (n = 54)				
	No	16 (53.3)	8 (33.3)	0.232**
	Yes	14 (46.7)	16 (66.7)	
Tocilizumab (n = 154)				
	No	69 (65.7)	18 (36.7)	**0.001** **
	Yes	36 (34.3)	31 (63.3)	

* Column percentage,

** Pearson chi-squared test.

**Table 4:** Regression analysis of the factors affecting the development of infection.

All patients *	Factors	p-value	OR	95% CI
	Age	0.865	-	-
	Sex: male	0.961	-	-
	Treatment with anakinra	0.499	-	-
	ICU (+)	0.001	32.819	4.382–245.821
	Length of hospital stay	0.004	1.048	1.015–1.082
Risk factor analysis of infection				
Patients followed-up in the ICU**	Factors	p-value	OR	95% CI
	Age	0.727	-	-
	Sex: male	0.979	-	-
	Treatment with anakinra	0.462	-	-
	Length of hospital stay	0.004	1.048	1.015–1.082

* Hosmer and Lemeshow test: p = 0.356, Cox & Snell R square value = 0.217, Nagelkerke R square value = 0.299. The model was significantly significant, χ2 (1, n = 208) = 51.010, p < 0.001, which suggests that it can distinguish between infection and noninfection. The model explained between 21.7% (Cox & Snell R square) and 29.9% (Nagelkerke R square) of the variance in the infection variable and the overall prediction of the classification was 69.2%.

** Hosmer and Lemeshow test: p = 0.271, Cox & Snell R square value = 0.067, Nagelkerke R square value = 090. The model was significantly significant, χ2 (1, n =160) = 11.122, p = 0.025, which suggests that it can distinguish between infection and noninfection. The model explained between 6.7% (Cox & Snell R square) and 9.0% (Nagelkerke R square) of the variance in the infection variable and the overall prediction of the classification was 59.4%.
